# Supplementary material for: RNA Structure in the 5′ Untranslated Region of Enterovirus D68 Strains with Differing Neurovirulence Phenotypes
Source: Viruses. 2023 Jan 20;15(2):295. doi: 10.3390/v15020295 (PMC9959730; doi:10.3390/v15020295)
Supplement: Supplementary file 1 [file viruses-15-00295-s001.zip › viruses-2117046-supplementary.pdf]

Instructions for viewing the .pdb 3D structures in S1 and S2

Figures S1 and S2 are .pdb files which contain the data necessary to visualize and interact with the tertiary structures of the Fermon and 2014 isolates of EV-D68 established here. These structures can be visualized quickly and easily through a variety of means. There are a variety of free visualization softwares such as PyMOL (https://pymol.org/2/), ChimeraX (http://www.cgl.ucsf.edu/chimera/), RasMol (bernstein-plus-sons.com/software/rasmol/) that can be downloaded and used to visualize the PDB files. There are also a variety of webservers that can be used to visualize the PDB files such as the PDB Mol 3D Viewers (https://www.rcsb.org/3d-view), Protein Imager (https://3dproteinimaging.com/protein-imager/), and EzMol (http://www.sbg.bio.ic.ac.uk/~ezmol/). A list of further visualization program can be found at https://www.rcsb.org/docs/additional-resources/molecular-graphics-software. Only the systems listed above have been tested to ensure that they can visualize these structures but and software capable of visualizing .pdb files should produce the same results.
